# Supplementary material for: Analytical Quality by Design (AQbD) Approach to the Development of Analytical Procedures for Medicinal Plants
Source: Plants (Basel). 2022 Nov 2;11(21):2960. doi: 10.3390/plants11212960 (PMC9653622; doi:10.3390/plants11212960)
Supplement: Supplementary file 1 [file plants-11-02960-s001.zip › plants-1968789 supplemently.pdf]

Table S1. Applications of AQbD to the development of analytical procedures for plant sources, published from 2016 to 2021.

Medicinal plant	ATP 1; APIs	ATP 2; Technique	ATP 3; Method requirements	Risk assessment	CAPAs	Potential APPs	CAPPs	DoE Screening	Optimization	MODR	Ref.
Ginkgo biloba	Terpene trilactones (ginkgolide A, B, and C, bilobalide)	HPLC-ESI-MS	Quantification	Ishikawa diagram	ion peak area	solvent flow rate; formic acid conc.; column temp.; gas flow rate; gas temp.; nebulizer pressure; capillary voltage	solvent flow rate; formic acid conc.; gas flow rate; gas temp.	PBD	BBD	Monte-Carlo probability	[41]
Plumbago species	Napthoquinone (plumbagin)	HPLC-PDA	Quantification	Ishikawa diagram (CMPs); Risk estimation matrix (CMAs)	retention time; peak area; symmetry; theoretical plates	N/A	plumbagin conc.; flow rate; solvent ratio	N/A	BBD	Desirability function	[37]
Daphne genkwa	Flavonoids (apigenin-5-*O*-glucosdie, apigenin-7-*O*-glucoside, yuanhuanin, apigenin-7-*O*-glucuronide, genkwanin-5-*O*-primeveroside, genkwanin-5-*O*-glucoside, genkwanin-4’-*O*-rutinoside, tiliroside, apigenin, 3’-hydroxy-genkwanin, genkwanin)	UHPLC-PDA	Separation; Quantification	Ishikawa diagram; FMEA	(screening) countable peak number; (optimization) sum of Rs	injection volume; column temp.; gradient slope (run time); flow rate	column temp.; gradient slope (run time)	FFD	CCD	N/A	[34]
Curcuma longa	Curcuminoids (curcumin, demethoxy-curcumin, bisdemethoxy-curcumin)	HPLC-UV	Quantification	N/A	symmetry; peak width	N/A	orthophosphoric acid conc.; solvent ratio	N/A	FFD	Overlay plot	[40]
Paeonia lactiflora *Angelica gigas*	Monoterpenoid (paeoniflorin) Courmarin (decursin)	HPLC-PDA	Quantification	Risk estimation matrix	critical Rs; symmetry	N/A	column temp.; gradient elution	N/A	CCD	Overlay plot	[36]
Cannabis species	Cannabinoids (cannabidiol, Δ9-tetrahydro-	UHPSFC-UV	Separation; Quantification	Ishikawa diagram	critical Rs	N/A	column temp.; column batch; formic acid conc.;	N/A	FFD	N/A	[43]

Medicinal plant	ATP 1; APIs	ATP 2; Technique	ATP 3; Method requirements	Risk assessment	CAPAs	Potential APPs	CAPPs	DoE Screening	Optimization	MODR	Ref.
	cannabinol, Δ9-tetrahydro-cannabinolic acid A, cannabidiolic acid, cannabinol, cannabigerol, cannabigerolic acid, Δ8-tetrahydro-cannabinol, cannabichromene)						starting gradient				
Sugarcane Honey	Sugars (D-glucose, D-fructose, sucrose, D-xylose, D-mannose, L-rhamnose)	HPLC-RI	Separation; Quantification	Ishikawa diagram	total peak area; critical Rs; symmetry; RSD	type of solvent; acetone conc.; flow rate; column temp.	acetone conc.; flow rate; column temp.	N/A	FFD	PRA; RSM; Monte-Carlo probability; Desirability function	[44]
Cannabis-olive oil	Cannabinoids (cannabidiol, Δ^9-tetrahydro-cannabinol)	HPLC-UV	Selective determination	Ishikawa diagram	analysis time; critical Rs	N/A	column temp.; pH; flow rate	N/A	Doehlert design	Bayesian probability	[74]
Glycyrrhiza glabra	Flavonoids (glucoliquiritin, schaftoside, isoliquiritin apioside, liquiritin apioside, liquiritin, neolicuroside, isoliquiritin, licorice glycoside A, liquiritigenin, Triterpenoids (licorice saponin A_3, 22-acetoxy-glycyrrhizin, licorice saponin G_2, glycyrrhizin acid)	UHPLC-PDA	Chromatographic fingerprint	Ishikawa diagram; FMEA	critical Rs; total peak number; capacity factor	gradient elution; flow rate; column temp.; wavelength; formic acid conc.	gradient elution	PBD	CCD	Monte-Carlo probability	[35]
Diospyros kaki	Polyphenols	UPLC-MS/MS	Selective determination	Ishikawa diagram	analysis time; critical Rs	flow rate; column temp.; starting organic phase	column temp.; starting organic phase conc.;	Asymmetric screening matrix	Doehlert design	Monte-Carlo probability	[42]

Medicinal plant	ATP 1;<br>APIs	ATP 2;<br>Technique	ATP 3;<br>Method requirements	Risk assessment	CAPAs	Potential APPs	CAPPs	DoE Screening	Optimization	MODR	Ref.
						conc.;<br>formic acid conc.;<br>type of organic solvent;<br>gradient elution	formic acid conc.;<br>gradient elution				
Codonopsis pilosula,<br><br>*Astragalus membranaceus*	Sugars<br>(D-glucose,<br>D-fructose,<br>sucrose)	HPLC-ELSD	Quantification	N/A	critical Rt;<br>S/N ratio	gradient elution;<br>flow rate;<br>column temp.;<br>gradient run time;<br>triethylamine conc.;<br>ELSD drift tube temp.;<br>gas flow rate	gradient elution;<br>flow rate;<br>column temp.	fFD	BBD	Monte-Carlo probability	[45]
Sugarcane honey (*Saccharum officinarum* L.)	Sugars<br>(5-hydroxymethyl-2-furaldehyde,<br>2-furaldehyde,<br>2-furylmethanol,<br>2-furyl methyl ketone,<br>5-methyl-2-furaldehyde)	MEPS-UHPLC-PDA	Separation;<br>Quantification	Ishikawa diagram	(MEPS)<br>total peak area;<br>RSD<br>(UHPLC-PDA)<br>total peak area;<br>critical Rs	N/A	(MEPS)<br>elution solvent;<br>loading cycles;<br>sample loading volume;<br>sorbent type<br>(UHPLC-PDA)<br>organic solvent;<br>flow rate;<br>column temp.;<br>column type	N/A	(MEPS)<br>FFD<br>(UHPLC-PDA)<br>FFD	Monte-Carlo probability;<br>Capability analysis	[46]
Coptis chinensis	Alkaloids<br>(jateorrhizine,<br>columbamine,<br>epiberberine,<br>coptisine,<br>palmatine,<br>berberine)	HPLC-PDA	Separation;<br>Short analysis time	N/A	(screening)<br>critical Rs;<br>analysis time;<br>peak width<br>(optimization)<br>critical Rs;<br>analysis time	gradient elution;<br>pH;<br>column temp.;<br>sodium dodecyl sulfate conc.;<br>potassium phosphate monobasic conc.	gradient elution;<br>sodium dodecyl sulfate conc.;<br>potassium phosphate monobasic conc.	PBD	BBD	Bayesian probability	[69]
Codonopsis pilosula<br><br>*Astragalus membranaceus*	Monosaccharide derivative<br>(syringin,<br>9,10-dimethoxy-ptercarpan-3-*O*-glucoside)<br>Flavonoids<br>(calycosin-7-*O*-glucoside,<br>ononin,<br>2'-hydroxy-3'4'-dimethoxyisoflavan-7-*O*-glucoside)<br>Polyacetylene	SPE-HPLC-UV/ELSD	Separation;<br>Quantification	preliminary experiments	critical Rs;<br>S/N	(SPE)<br>sorbent mass;<br>sample volume;<br>elution volume;<br>elution speed<br>(HPLC-UV/ELSD)<br>formic acid conc.;<br>flow rate;<br>column temp.;<br>evaporator temp.;<br>nebulizer temp.;<br>gas flow rate;<br>PMT gain	(SPE)<br>sorbent mass;<br>sample volume;<br>(HPLC-UV/ELSD)<br>flow rate;<br>column temp.;<br>evaporator temp.;<br>gas flow rate	PBD	BBD	Monte-Carlo probability	**[47]**

Medicinal plant	ATP 1; APIs	ATP 2; Technique	ATP 3; Method requirements	Risk assessment	CAPAs	Potential APPs	CAPPs	DoE Screening	Optimization	MODR	Ref.
	glycoside (lobetyolin) Triterpenoids (astragaloside IV, astragaloside II, astragaloside I)										
Panax notoginseng	Saponins (notoginsenoside R1, ginsenoside Rg1, ginsenoside Re, ginsenoside Rb1, ginsenoside Rd)	HPLC-DAD	Separation; Quantification	N/A	analysis time; critical Rs; symmetry	column temp.; flow rate; gradient elution	gradient elution	Screening design	fFD	Monte-Carlo probability; Capability analysis	[38]
Panax notoginseng	Saponins (notoginsenoside R1, ginsenoside Rg1, ginsenoside Re, ginsenoside Rb1, ginsenoside Rd)	HPLC-UV	Separation; Quantification	N/A	critical Rs	N/A	gradient elution	N/A	CCD	Monte-Carlo probability	[39]

Active pharmaceutical ingredient (API); analytical target profile (ATP); Box-Behnken design (BBD); central composite design (CCD); critical analytical procedure attribute (CAPA); analytical procedure parameter (APP); critical analytical procedure parameter (CAPP); concentration (conc.); design of experiment (DoE); evaporative light-scattering detector (ELSD); electrospray ionization (ESI); full factorial design (FFD); fractional factorial design (fFD); failure mode effect analysis (FMEA); high-performance liquid chromatography (HPLC); micro-extraction by packed sorbent (MEPS); method operable design region (MODR); mass spectrometry (MS); not applicable (N/A); Plackett-Burman design (PBD); photodiode array (PDA); pareto ranking analysis (PRA); refractive index (RI); risk priority number (RPN); resolution (Rs); relative standard deviation (RSD); response surface methodologies (RSM); retention time (RT); signal to noise (S/N); solid phase extraction (SPE); temperature (temp.); ultra-high-performance supercritical fluid chromatography (UHPSFC); ultra-high-performance liquid chromatography (UHPLC); ultraviolet (UV).
